# Modified Suanzaorentang Had the Treatment Effect for Generalized Anxiety Disorder for the First 4 Weeks of Paroxetine Medication: A Pragmatic Randomized Controlled Study

**DOI:** 10.1155/2017/8391637

**Published:** 2017-05-03

**Authors:** Ming-Fen Song, Lin-Lin Hu, Wen-Juan Liu, Yi Liu, Xiao-Yun Tao, Ting-Ting Wang, Sheng-Dong Wang, Long Zhang, Yong-Hua Zhang

**Affiliations:** ^1^Internal Medicine of Traditional Chinese Medicine, The 2nd Clinical Medical College, Zhejiang Chinese Medical University, 548 Binwen Road, Hangzhou, Zhejiang 310053, China; ^2^Molecular Biology Laboratory, Hangzhou Seventh People's Hospital, 305 Tianmushan Road, Hangzhou, Zhejiang 310013, China; ^3^Psychosomatic Disorders Department, Hangzhou Seventh People's Hospital, 305 Tianmushan Road, Hangzhou, Zhejiang 310013, China

## Abstract

*Background*. Paroxetine does not show satisfactory therapeutic effect for generalized anxiety disorder (GAD) patients for the first 2–4 weeks of medication. Diazepam is always concurrently used although it has some shortcomings such as physical dependence and withdrawal reactions. In this study, we aimed to identify whether modified Suanzaorentang (MSZRT), a combined Chinese formula including Suanzaorentang (SZRT) and Zhizichitang (ZZCT), could control the anxiety of GAD for the first 4 weeks of paroxetine medication.* Methods*. 156 GAD patients were randomized to the treatment of paroxetine, paroxetine-diazepam, or paroxetine-MSZRT for 4 weeks. Hamilton Anxiety Scale (HAMA) Test and Self-Rating Anxiety Scale (SAS) Test were determined each week as the evaluation of clinical efficacy. Adverse events (AEs) were also closely observed by performing the Treatment Emergent Symptom Scale (TESS) Test.* Results*. Both paroxetine-MSZRT and paroxetine-diazepam decreased more HAMA and SAS total scores than paroxetine from weeks 1 to 3. Paroxetine-MSZRT as well as paroxetine-diazepam had an obviously higher onset rate than paroxetine in each week. After 4 weeks' treatment, the overall effectiveness rate in the paroxetine-MSZRT group (90.00%) was obviously higher than those of the paroxetine group (74.42%) but did not significantly differ from the paroxetine-diazepam group (93.88%).* Conclusion*. MSZRT had the treatment effect for GAD when paroxetine was used for the first 4 weeks.

## 1. Introduction

Generalized anxiety disorder (GAD) is one of the common neuroses with lifetime prevalence rate of 4–7% in general population [1, 2] and characterized by excessive, uncontrollable, and often irrational worry [2–4]. Chemical drugs have been widely used for the treatment of GAD worldwide. For example, benzodiazepines, which enhance the effect of the neurotransmitter gamma-aminobutyric acid (GABA) via targeting GABA_A_ receptor, are classic anxiolytics for GAD [5, 6]. But benzodiazepines are not recommended for a long-term treatment, because they are associated with tolerance, psychomotor impairment, cognitive and memory changes, physical dependence, and withdrawal reaction on discontinuation [7]. However, 40% of GAD patients had reported illness duration lasting >5 years [8] and a long-term efficacious and safe protocol is required [9, 10].

In recent years, paroxetine is widely used for the long-term continuing treatment of GAD [8, 11]. However, its anxiolytic response in GAD patients represents an obviously delayed process [12]. This delayed response causes patients to doubt paroxetine treatment and then decrease their compliance. In order to control anxiety as soon as possible, benzodiazepines such as diazepam are still concurrently given with paroxetine for a short time although they have some shortcomings mentioned above.

Chinese herbal medicine, as one of the most popular complementary therapies of Western medicine, usually in herbal formula, has been commonly used and widely accepted. Recently, our research group has been trying to establish an alternative to benzodiazepines by using Chinese herbal medicine when paroxetine is used for GAD treatment.

Suanzaorentang (SZRT), a formula of five medicinal Chinese herbs including Semen Zizyphi Spinosae (Suanzaoren), Sclerotium Poriae Cocos (Fuling), Radix Ligustici Chuanxiong (Chuanxiong), Rhizoma Anemarrhena (Zhimu), and Radix Glycyrrhizae (Gancao), is beneficial for the replenishment of yin and proposed to have the tranquillizing effect according to* Jin Gui Yao Lve* written by Zhong-Jing Zhang in approximately 210 AD. Thus, SZRT was selected as the basic Chinese medicine for the treatment of GAD in this study. Besides SZRT, we added Zhizichitang (ZZCT), which was also documented in* Jin Gui Yao Lve*, comprising Zhizi (*Gardenia jasminoides* fruit), Dandouchi (Fermented Soybean), Chanyi (periostracum cicada), and aids in eliminating internal fire. We named this new combined formula of SZRT and ZZCT as modified SZRT (MSZRT) in this study. Yin-deficiency and fire-excess syndrome are common in GAD patients and often exhibit somatic symptoms such as insomnia, palpitation, restlessness, headache, and rapid pulse, flash, and excessive sweating. Therefore, the combination of these two classic compounds may perfectly complement each other in sedative effect for GAD patients. Based on our experience, we predicted that MSZRT may display its sedative effect right after medication. However, the evidence about the treatment efficacy and safety of MSZRT on GAD patients is still lacking.

In the present study, we aimed to identify whether MSZRT could control the anxiety and solve the delayed therapeutic effect when paroxetine is used for GAD patients. 156 subjects were recruited and randomly assigned to receive the treatment of paroxetine, paroxetine-diazepam, or paroxetine-MSZRT. The treatment efficacy was compared among groups. In addition, adverse events (AEs) were also closely observed to ensure the tolerance of MSZRT.

## 2. Materials and Methods

### 2.1. Recruitment of Subjects

This study was approved by the Ethics Committee of Hangzhou Seventh People's Hospital. The written informed consent from each subject was obtained before study. They were allowed and free to withdraw from this study for any reasons at any time.

First of all, inpatients of Psychosomatic Disorders Department in our hospital, diagnosed as GAD by two experienced psychiatrists based on DSM-V and treatment-free within 2 months, were recruited. Next, patients were estimated with Hamilton Anxiety Scale (HAMA) Test by a trained clinician and Self-Rating Anxiety Scale (SAS) by themselves. In this study, participants were required to have a score ≥ 14 on HAMA and ≥50 on SAS at baseline.

It was reported that GAD patients with a depressive episode, either of MDD or of BPD, can also have a high HAMA total score [12]. To purify the subjects, we also determined Hamilton depression scale (HAMD) for each participant and those with score ≥ 7 were excluded in the present study. Furthermore, those patients with evidence of drug abuse, drinking, cognitive impairment, and physical illness such as diabetes, severe hypertension, cardiovascular and cerebrovascular diseases, malignant diseases, respiratory diseases, or autoimmune infections were also excluded.

156 subjects (69 men and 87 women) from January, 2015, to March, 2016, meeting our inclusion and exclusion criteria, were recruited. All subjects were randomly assigned to receive the treatments of paroxetine, paroxetine-diazepam, or paroxetine-MSZRT. Finally, 14 subjects quit this study due to serious AEs or anxiety deterioration during the whole study period (9: anxiety deterioration from the paroxetine group; 1: dizziness, 2: constipation from the paroxetine-diazepam group; 2: diarrhea from the paroxetine-MSZRT group). Finally, 43 cases (17 men and 26 women) in the paroxetine group, 49 cases (21 men and 28 women) in the paroxetine-diazepam group, and 50 cases (22 men and 28 women) in the paroxetine-MSZRT group were analyzed.

### 2.2. Drug Preparation

Daily dose of MSZRT formula for each patient comprised Suanzaoren (Semen Zizyphi Spinosae) 15 g, Zhimu (Rhizoma Anemarrhena) 12 g, Fuling (Sclerotium Poriae Cocos) 15 g, Chuanxiong (Radix Ligustici Chuanxiong) 10 g, Zhizi (*Gardenia jasminoides* fruit) 10 g, Dandouchi (Fermented Soybean) 6 g, Chanyi (periostracum cicada) 6 g, and Zhigancao (Radix Glycyrrhizae) 6 g. All herbs were purchased from Medicinal Materials Co. Ltd. (Lin'an City, Zhejiang Province, China). They were mixed and prepared as 400 ml of decoction solution according to traditional methods and packed into two bags. Paroxetine (20 mg/tablet) was obtained from Tianjin Smith Kline & French laboratories Ltd., China. Diazepam (2.5 mg/tablet) was purchased from Beijing Yimin Pharmaceutical Co., Ltd., China.

### 2.3. Patient Treatments

Subjects in three groups took paroxetine 20 mg/day half an hour after breakfast in the first week. From second week, they were allowed to increase paroxetine dose. The maximum dose during the study period was 60 mg/day if judged clinically necessary by the investigator. Meanwhile, the paroxetine-diazepam group received 2.5 mg of diazepam three times daily as recommended by the manufacturer. The paroxetine-MSZRT group received two bags of the MSZRT decoction per day and drank them half an hour after breakfast and supper based on the traditional administration method for Chinese herbal formula. No other medications or psychotherapy were permitted during study period.

### 2.4. Efficacy Evaluation

HAMA total scores at baseline and weeks 1, 2, 3, and 4 after treatment were evaluated as the primary outcome measurement by a trained clinician, who was blind to the treatment for each patient. Subjects also performed SAS test at all observation points as the secondary outcome measurement to confirm the results obtained from HAMA test.

When the reduction rate of HAMA total score first reached ≥25% as compared with baseline, the treatment onset was considered.

At the end of the observation, we judged the treatment efficacy for each participant based on the reduction rate of HAMA total score compared to baseline. The reduction rate ≥ 75% was considered as clinical control, 50–75% was regarded as marked effectiveness, 25–50% was viewed as effectiveness, and <25% was defined as ineffectiveness. Thus, when study is finished, we set a reduction rate ≥ 25% in the HAMA total score as overall effectiveness and <25% as ineffectiveness. We also considered HAMA total score ≤ 7 at the final observation as clinical remission. The overall effectiveness rate and clinical remission rate were calculated for each group.

### 2.5. Estimation of AEs

Throughout the study, the subjects were monitored closely for AEs. We used the Treatment Emergent Symptom Scale (TESS) to evaluate AEs including behavioral toxicity, laboratory examination, nerve system, autonomic nervous system, cardiovascular system, and others such as skin symptom, body weight, headache, and appetite. The value for each item on the scale ranged from zero to four: zero meant no AE, one indicated mild AE, two represented moderate AE, three showed severe AE, and four expressed very severe AE.

### 2.6. Statistical Analysis

All statistical analyses were conducted using SPSS 19.0 software package (SPSS Inc., America). Baseline demographic characteristics were compared by using independent sample ANOVA for continuous variables and *χ*^2^ test for categorical variables. Repeated measurement ANOVA test was carried out for the comparisons of HAMA or SAS total scores at all observation points among three groups. *χ*^2^ test was performed for the comparisons of onset rates, overall effectiveness rates, and clinical remission rates among three groups. Kruskal-Wallis *H* test followed by Nemenyi test was run to analyze the distribution difference of treatment efficacy among three groups.

## 3. Results

### 3.1. Baseline Characteristics

The information about sex, marriage, age, BMI, and education of all analyzed subjects was collected and shown in Table 1. No significant difference in all demographic characters was observed among the three groups (*P* > 0.05). All subjects included in the present study were nonsmokers and nondrinkers to avoid their possible influences.

The doses of paroxetine were (40.40 ± 8.80) mg in the paroxetine group, (38.98 ± 7.70) mg in the paroxetine-diazepam group, and (38.00 ± 9.69) mg in the paroxetine-MSZRT group. They were not statistically different among the three groups (*F* = 0.62, *P* = 0.54).

### 3.2. Comparison of HAMA Total Scores

**Figure 1:**
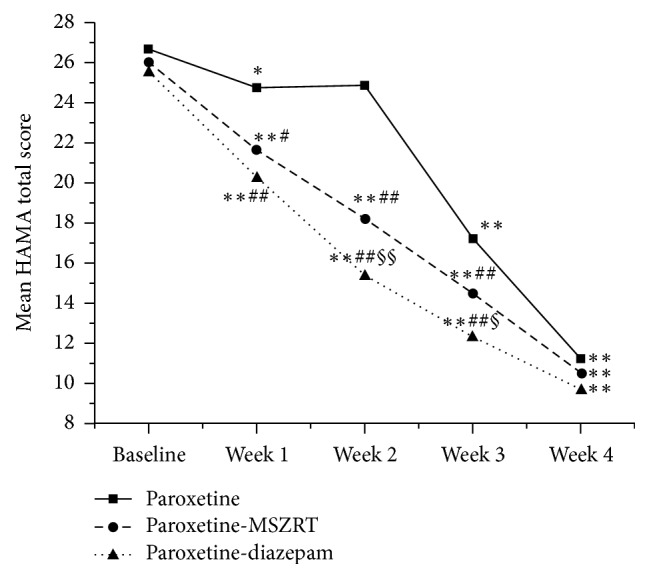
The mean HAMA total scores among the three groups. *∗* means *P* < 0.05 and *∗∗* depicts *P* < 0.01 as compared to the former weeks in each group. # means *P* < 0.05 and ## depicts *P* < 0.01 as compared to the paroxetine group. § means *P* < 0.05 and §§ depicts *P* < 0.01 as compared to the paroxetine-MSZRT group.

The change of HAMA total scores in three groups was shown in Figure 1. At week 1, as compared to those of baseline, the paroxetine group had slightly decreasing effect on HAMA total score (from 26.66 to 24.72, *P* < 0.05), while two combined treatments exhibited obviously decreasing effect (from 26.02 to 21.63 in the paroxetine-MSZRT group, from 25.48 to 20.20 in the paroxetine-diazepam group, both *P* < 0.01). At week 2, the paroxetine group did not show a statistical change in HAMA total score while two combined groups were found to have a significant decrease (both *P* < 0.01) as compared to those of week 1. At weeks 3 and 4, each group had a continuous decrease in HAMA total score as compared with their former weeks (all *P* < 0.01). Paroxetine-diazepam seemed to decrease HAMA scores more than paroxetine-MSZRT at weeks 2 and 3 (*P* < 0.01 or *P* < 0.05). Following treatment for 4 weeks, no obvious difference in HAMA total scores was observed among the three groups (*P* > 0.05).

### 3.3. Comparison of Onset

The treatment onset in each group was shown in Table 2. Both paroxetine-MSZRT and paroxetine-diazepam had obviously higher onset rates than paroxetine alone from weeks 1 to 4 (all *P* < 0.05), especially at weeks 1 and 2. However, the difference between paroxetine-MSZRT and paroxetine-diazepam did not reach statistical significance (*P* > 0.05).

### 3.4. Comparison of SAS Scores

**Figure 2:**
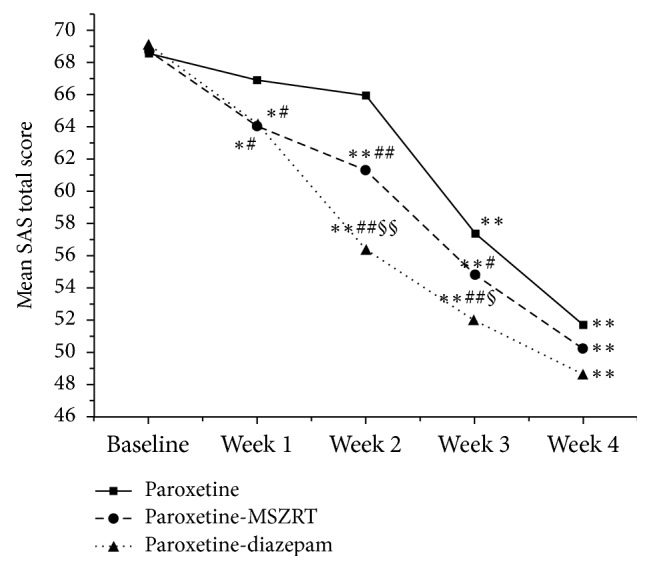
The mean SAS total scores among the three groups. *∗* means *P* < 0.05 and *∗∗* depicts *P* < 0.01 as compared to the former weeks in each group. # means *P* < 0.05 and ## depicts *P* < 0.01 as compared to the paroxetine group. § means *P* < 0.05 and §§ depicts *P* < 0.01 as compared to the paroxetine-diazepam group.

The results of self-reported SAS scores were similar to clinician-rated HAMA scores (Figure 2). In brief, paroxetine did not show obvious therapeutic effect at week 1 (*P* = 0.13) and week 2 (*P* = 0.22) as compared with baseline. From weeks 3 and 4, a significant decrease of SAS score was observed in this group (*P* < 0.01 comparing week 3 versus week 2 and week 4 versus week 3). However, paroxetine-diazepam and paroxetine-MSZRT kept decreasing SAS scores from week 1 to week 4 (*P* < 0.01). Paroxetine-diazepam was also observed to decrease SAS scores more than paroxetine-MSZRT at weeks 2 and 3 (*P* < 0.01 or *P* < 0.05). At the final observation, there was no statistical difference among the three groups (*F* = 2.61, *P* = 0.08).

### 3.5. Comparison of Treatment Efficacy

The treatment efficacy in the three groups was showed in Table 3.

The overall effectiveness rate was 74.42%, 93.88%, and 90.00% for the paroxetine, paroxetine-diazepam, and paroxetine-MSZRT group, respectively. Although the two combined treatment groups did not have obvious difference in overall effectiveness rate (*χ*^2^ = 0.50, *P* = 0.48), they were significantly higher than that of the paroxetine group (*χ*^2^ = 5.54, *P* = 0.02 for the paroxetine-diazepam group; *χ*^2^ = 3.94, *P* = 0.047 for the paroxetine-MSZRT group). After 4 weeks' treatment, the distribution of overall effectiveness among the three groups was not statistically different (*χ*^2^ = 0.48, df = 2, *P* = 0.79).

The clinical remission rates in the three groups were 9.30%, 12.24%, and 10.00%. There was no significant difference among the three groups (*χ*^2^ = 0.24, *P* = 0.89).

### 3.6. Evaluation of AEs

The subjects in this study reported AEs mainly in gastrointestinal system and some feelings such as drowsiness, dizziness, and headache as shown in Table 4. We did not calculate the mean score of each item because most of them were evaluated as 0. The incidence rates of nausea, loss of appetite, diarrhea, constipation, drowsiness, dizziness, headache, and sexual dysfunction were not significantly different among the three groups (*P* > 0.05).

No abnormal laboratory changes were observed in any of the patients from the initial screen to the last evaluation.

## 4. Discussion

In the present clinical trial, we determined whether MSZRT could control the anxiety and solve the delayed therapeutic response when paroxetine is used for GAD patients for the first 4 weeks of medication.

Clinical trial can be designed to be either pragmatic or explanatory [13]. Explanatory trials are designed to find out whether a treatment has any efficacy (usually compared with placebo) under ideal, experimental conditions [13]. However, it is a big challenge to perform an explanatory clinical trial in the study of Traditional Chinese Medicine due to the fact that a suitable solution as the placebo of Chinese herbal medicine is still lacking. Fortunately, a widely accepted study design, pragmatic clinical trial, has been developed for this situation. Pragmatic trials are designed to find out about how effective a treatment actually is in routine, everyday practice. They are used to test an overall “package” of care, including the contribution of the therapeutic relationship, patients' expectations, and any specific therapy that is used. They compare the effect of this package of care with another treatment, not with a placebo. Thus, the pragmatic design is especially useful where the use of a placebo control to separate specific from nonspecific effects is problematic. In addition, blindness in this type of study is not as strict as explanatory trial. Participants are allowed to know what are the treatments they receive [13].

Our present study is a pragmatic trial, which exactly reflects our everyday clinical practice. The reasons why we designed this study as a pragmatic trial are as follows. First, we could not find a satisfactory solution as the placebo for MSZRT, which is similar to MSZRT in color, taste, and smell. Second, we wanted this study to reflect the routine treatment of Chinese herbal medicine in the medication method. Recently, some researchers manufactured the extract of Chinese herbal medicine into powder or granules, while their placebo was prepared with starch or artificial pigments in the same form and similar color, smell, and taste [14, 15]. It was a great way to solve the problem of placebo. However, we have never used powder or granules of MSZRT in our clinical practice before. Because powder or granules may present different treatment effects from decoction solution based on the Traditional Chinese Medicine theory. The decoction solution drinking is believed to be the best way to exert treatment effects of Chinese herbal medicine. That is why the decoction solution has been used for the medication method for a long history about thousands of years and it still is the most classical and widely accepted method in Traditional Chinese Medicine. Thus, in this study, we chose the decoction solution of MSZRT for the paroxetine-MSZRT group in order to keep the same medication method between research and clinical real use.

Although paroxetine is an efficacious approach for GAD, it has obvious delayed therapeutic onset. A study showed that the effect of paroxetine was seen in patients with somatic anxiety after 3-4 weeks and in patients with cognitive anxiety after 3–6 weeks [16]. The mechanism of this delayed effect is still unknown. It was reported that the steady state levels of paroxetine in body were achieved after 4–14 days of medication [17]. The time required for steady levels of paroxetine to exert their full effects through a reaction cascade after drug intake was considered to contribute the delayed effect of paroxetine [17]. On the other hand, 5-HT_1A_ autoreceptors were also thought to be involved. It was reported that 5-HT_1A/1B_ −/− mice induced a strong anxious-like behavioral state [18]. The 5-HT_1A_ receptor antagonist pindolol had been combined with SSRIs in patients with anxiety disorders to shorten the onset of the clinical action and increase the proportion of responders [19]. It was believed that serotonergic negative feedback mediated by 5-HT_1A_ autoreceptors to decrease the synthesis and release of 5-HT after SSRI medication was associated with the delayed therapeutic effect [20–22]. Thus, 5-HT_1A_ autoreceptors desensitization was required before SSRI exerting effect [23]. In the present study, we also observed a deterioration of anxiety during paroxetine treatment in some cases. Its cause or mechanism remains unknown. A paper reported that 5-HT_2A_ receptors were considered to be involved and their activation may attenuate paroxetine-induced anxiety [24].

In our previous study, we found that SZRT together with Zhi Zi Chi Tang, which was the same formula as MSZRT in the present study, could decrease SAS scores and improve daytime function in insomniacs with anxiety [25]. Insomnia is one of the common symptoms of GAD [26] or it is frequently cooccurring [27]. In our current study, we hypothesized that MSZRT may also have treatment effect for GAD due to its role of tranquillization according to TCM theory. Our results showed that paroxetine-MSZRT decreased HAMA and SAS total scores obviously during the study period (Figures 1 and 2). In addition, paroxetine-MSZRT had obviously higher onset rates (Table 2) and overall effectiveness rates (Table 3) than paroxetine. These suggested that MSZRT had the ability to control the anxiety of GAD for the first 4 weeks of paroxetine medication. However, paroxetine-MSZRT showed less effective actions in decreasing HAMA and SAS total scores as compared to those of paroxetine-diazepam at weeks 2 and 3. This may be due to the fact that Chinese herbal formula focuses on adjusting system balance in body and always shows moderate role for disease treatment.

The mechanism of MSZRT on GAD treatment is still unknown. It was reported that the effect of SZRT may be associated with serotonergic system based on the fact that SZRT exhibited binding affinity for serotonin receptors [28] and the sleep regulation effect of SZRT was blocked via using 5-HT_1A_, 5-HT_2_, and 5-HT_3_ antagonists [29]. GABAergic system was also considered to be involved in the mechanism of SZRT effect through GABA_A_ receptor associated chloride channel [30]. In addition, Yang et al. reported that the components of amino acid and fatty acid in SZRT would also be in response to the treatment effect through immune and nervous system [28]. Although ZZCT is also a common Chinese formula used for sedation and has the synergistic effect together with SZRT, its underlying mechanism with/without SZRT has seldom been studied. Thus, further researches to elucidate the possible mechanism of MSZRT for GAD treatment are needed.

The most AEs in the paroxetine-MSZRT group were some mild gastrointestinal reactions such as nausea, diarrhea, anorexia, dry mouth, and some whole body feelings such as drowsiness, dizziness, and headache, which were also observed in the other two groups of this study and reported by other authors previously. For example, Lai et al. found that dizziness, headache, stomach ache, and diarrhea were probably related to SZRT treatment [31]. In this study, we did not find any significant difference in the incidence of each AE item among three groups (Table 4). These results suggested that MSZRT was generally well tolerated for GAD patients. Sexual side effect is commonly associated with SSRI treatment. Some evidence indicated that the activation of 5-HT_1A_ receptors mitigated SSRI-induced sexual dysfunction [32, 33]. However, MSZRT seemed to have no alleviation effect on sexual side effect in the present study (Table 4).

However, our study has limitations. First, MSZRT treatment effect included some placebo effect and we could not exclude this part in the analysis of data. This is the main limitation of a pragmatic design as compared with an explanatory design. Second, in order to protect patients' right to receive effective treatments and avoid conflict with the ethics, we did not use MSZRT alone to treat GAD patients due to its uncertain effect so far. Third, this study was carried out for only 4 weeks because the delayed effect of paroxetine usually occurs within 4 weeks and if paroxetine still did not work after 4 weeks' medication, the treatment protocol should be changed according to the Ethics Committee of our hospital. Fourth, we have not performed the mechanism study of MSZRT on GAD treatment in the present study. Further investigations with placebo, MSZRT-alone treatment, longer observation, and the underlying mechanism of MSZRT are required.

## 5. Conclusions

Our results suggested that MSZRT exhibited the anxiety-controlling effect for GAD by decreasing HAMA and SAS total scores, enhancing onset rate and overall effectiveness rate for the first 4 weeks of paroxetine treatment, which was also observed when diazepam was used. Thus, we recommend MSZRT as an alternative to diazepam and concurrent use of MSZRT and paroxetine as a new protocol for the treatment of GAD during this period.

## Figures and Tables

**Table 1:** The demographic characteristics of subjects.

Variables	Paroxetine	Paroxetine-diazepam	Paroxetine-MSZRT	Statistical analysis	
				*F*/*χ*^2^ value	*P* value
Number of subjects (*n*)	43	49	50		
Sex (male/female)	17/26	21/28	22/28	0.20	0.91
Marriage (married/single)	32/11	29/20	35/15	2.63	0.27
Age in year	50.60 ± 12.84	47.94 ± 12.10	48.96 ± 12.87	0.56	0.57
BMI (kg/m^2^)	21.82 ± 2.49	21.12 ± 2.30	20.94 ± 2.15	2.00	0.14
Education (year)	14.34 ± 4.34	13.69 ± 4.75	14.74 ± 4.92	0.63	0.53

Data are expressed as mean ± standard deviation.

**Table 2:** The onset case numbers in each week.

Onset numbers	Week 1	Week 2	Week 3	Week 4
	*n* (%)	*n* (%)	*n* (%)	*n* (%)
Paroxetine	3 (6.98%)	6 (13.95%)	27 (62.79%)	32 (74.42%)
Paroxetine-diazepam	41 (83.67%)^a^	36 (73.47%)^d^	44 (89.80%)^g^	46 (93.88%)^j^
Paroxetine-MSZRT	34 (68.00%)^b,c^	39 (78.00%)^e,f^	43 (86.00%)^h,i^	45 (90.00%)^k,l^

At week 1, ^a^*χ*^2^ = 53.99 and *P* = 0.000; ^b^*χ*^2^ = 35.94 and *P* = 0.000 as compared with the paroxetine group. ^c^*χ*^2^ = 3.31 and *P* = 0.07 as compared with the paroxetine-diazepam group.

At week 2, ^d^*χ*^2^ = 32.70 and *P* = 0.000; ^e^*χ*^2^ = 37.92 and *P* = 0.000 as compared with the paroxetine group. ^f^*χ*^2^ = 0.28 and *P* = 0.60 as compared with the paroxetine-diazepam group.

At week 3, ^g^*χ*^2^ = 9.48 and *P* = 0.002; ^h^*χ*^2^ = 6.69 and *P* = 0.01 as compared with the paroxetine group. ^i^*χ*^2^ = 0.34 and *P* = 0.56 as compared with the paroxetine-diazepam group.

At week 4, ^j^*χ*^2^ = 6.72 and *P* = 0.01; ^k^*χ*^2^ = 3.94 and *P* = 0.047 as compared with the paroxetine group. ^l^*χ*^2^ = 0.50 and *P* = 0.48 as compared with the paroxetine-diazepam group.

**Table 3:** The treatment efficacy among three groups.

Efficacy	Paroxetine	Paroxetine-diazepam	Paroxetine-MSZRT
	*n* (%)	*n* (%)	*n* (%)
*n*	43	49	50
Overall effectiveness	32 (74.42%)	46 (93.88%)^a^	45 (90.00%)^b,c^
Clinical control	7 (16.28%)	7 (14.29%)	9 (18.00%)
Marked effectiveness	20 (46.51%)	27 (55.10%)	26 (52.00%)
Effectiveness	5 (11.63%)	12 (24.49%)	10 (20.00%)
Ineffectiveness	11 (25.58%)	3 (6.12%)	5 (10.00%)
Clinical remission	4 (9.30%)	6 (12.24%)^d^	5 (10.00%)^e,f^

^a^*χ*^2^ = 5.54, *P* = 0.02 as compared with the paroxetine group.

^b^*χ*^2^ = 3.94, *P* = 0.047 as compared with the paroxetine group.

^c^*χ*^2^ = 0.50, *P* = 0.48 as compared with the paroxetine-diazepam group.

^d^*χ*^2^ = 0.21, *P* = 0.65 as compared with the paroxetine group.

^e^*χ*^2^ = 0.01, *P* = 0.91 as compared with the paroxetine group.

^f^*χ*^2^ = 0.13, *P* = 0.72 as compared with the paroxetine-diazepam group.

**Table 4:** Adverse events in the three groups.

Groups	Nausea	Loss of appetite	Diarrhea	Constipation	Drowsiness	Dizziness	Headache	Sexual dysfunction
	*n* (%)	*n* (%)	*n* (%)	*n* (%)	*n* (%)	*n* (%)	*n* (%)	*n* (%)
Paroxetine	6 (13.95%)	10 (23.26%)	4 (9.30%)	13 (30.23%)	11 (25.58%)	10 (23.26%)	5 (11.63%)	7 (16.28%)
Paroxetine-diazepam	6 (12.24%)	6 (12.24%)	3 (6.12%)	17 (34.69%)	13 (26.53%)	9 (18.37%)	6 (12.24%)	6 (12.24%)
Paroxetine-MSZRT	5 (10.00%)	7 (14.00%)	6 (12.00%)	15 (30.00%)	9 (18.00%)	6 (12.00%)	2 (4.00%)	8 (16.00%)
*χ* ^2^	0.35	2.32	1.03	0.31	1.20	2.05	2.48	0.39
*P* value	0.84	0.31	0.60	0.86	0.55	0.36	0.29	0.83
